# COVID-19 clinical outcomes and nationality: results from a Nationwide registry in Kuwait

**DOI:** 10.1186/s12889-020-09490-y

**Published:** 2020-09-10

**Authors:** Hala Hamadah, Barrak Alahmad, Mohammad Behbehani, Sarah Al-Youha, Sulaiman Almazeedi, Mohannad Al-Haddad, Mohammad H. Jamal, Salman Al-Sabah

**Affiliations:** 1 Johns Hopkins Bloomberg School of Public Health, Baltimore, MD USA; 2 Jaber Al-Ahmad Al-Sabah Hospital, Ministry of Health, Kuwait City, Kuwait; 3 Department of Environmental Health, Harvard T.H. Chan School of Public Health, Boston, MA USA; 4 Faculty of Medicine, Kuwait University, Kuwait City, Kuwait

**Keywords:** Migrant workers, Kuwait, COVID-19, Nationality, Health disparity

## Abstract

**Background:**

In light of the COVID-19 pandemic, many have flagged racial and ethnic differences in health outcomes in western countries as an urgent global public health priority. Kuwait has a unique demographic profile with two-thirds of the population consisting of non-nationals, most of which are migrant workers. We aimed to explore whether there is a significant difference in health outcomes between non-Kuwaiti and Kuwaiti patients diagnosed with COVID-19.

**Methods:**

We used a prospective COVID-19 registry of all patients (symptomatic and asymptomatic) in Kuwait who tested positive from February 24th to April 20th, 2020, collected from Jaber Al-Ahmad Al-Sabah Hospital, the officially-designated COVID-19 healthcare facility in the country. We ran separate logistic regression models comparing non-Kuwaitis to Kuwaitis for death, intensive care unit (ICU) admission, acute respiratory distress syndrome (ARDS) and pneumonia.

**Results:**

The first 1123 COVID-19 positive patients in Kuwait were all recruited in the study. About 26% were Kuwaitis and 73% were non-Kuwaiti. With adjustments made to age, gender, smoking and selected co-morbidities, non-Kuwaitis had two-fold increase in the odds of death or being admitted to the intensive care unit compared to Kuwaitis (OR: 2.14, 95% CI 1.12–4.32). Non-Kuwaitis had also higher odds of ARDS (OR:2.44, 95% CI 1.23–5.09) and pneumonia (OR: 2.24, 95% CI 1.27–4.12).

**Conclusion:**

This is the first study to report on COVID-19 outcomes between Kuwaiti and non-Kuwaiti patients. The current pandemic may have amplified the differences of health outcomes among marginalized subpopulations. A number of socioeconomic and environmental factors could explain this health disparity. More research is needed to advance the understanding of policymakers in Kuwait in order to make urgent public health interventions.

## Background

A new strain of the coronavirus, Severe Acute Respiratory Syndrome Coronavirus 2 (SARS-CoV-2), first appeared in Wuhan, China in mid-December of 2019 [1]. Over the following months, it has rapidly spread across the globe, resulting in a pandemic that countries have struggled to contain [1]. The most common clinical manifestations of COVID-19 include fever, sore throat, cough and muscle ache. COVID-19 can also result in severe respiratory symptoms and has led to over 350,000 direct deaths worldwide [1, 2]. Certain risk factors like old age, gender, diabetes, asthma, cardiovascular disease, and recent malignancy are all associated with increased mortality in patients [3].

Although data on the effect of racial and ethnic differences as related to COVID-19 remains scarce, some observations have been made in certain populations worldwide. In the United States, African Americans in Chicago, Illinois have accounted for about 70% of COVID-19 associated deaths whilst only accounting for 30% of Chicago’s population and 50% of all COVID-19 infections clustering in only five neighborhoods [4]. A recent retrospective study conducted in Louisiana revealed a higher proportion of African Americans being hospitalized and dying from COVID-19 [5]. In New York State, African American and Hispanic people had higher rates of mortality from COVID-19 than for White people [6]. In the United Kingdom, the Office of National Statistics reported that black males were 4.2 times more likely to die from COVID-19 compared to white males [7]. Many have flagged these early results as an urgent public health research priority [8, 9].

There is roughly about 150 million international migrant workers [10]. Prior to the start of COVID-19, access to healthcare for these international migrant workers was at times troublesome. Many of these workers, especially domestic workers, were less likely to have medical insurance and usually worked in countries that did not share their language. Public health service messaging in the times of COVID-19 therefore would prove to be an issue and reaching these communities whether to provide medical supplies like masks or to disseminate accurate information was a problem during this pandemic [10].

Kuwait, a small country in the Middle East with a population of 4.2 million, has also been affected by this disease [11]. Around two-thirds of Kuwait’s population is made up of non-Kuwaitis, most of whom are from other Arab nations, such as Egypt, Syria and Lebanon, or from Southeast Asian nations including India, Bangladesh, Pakistan and the Philippines. The majority of these non-Kuwaitis are young, male migrant workers that tend to work in construction, agriculture, fishery, and as domestic workers [12]. The first confirmed COVID-19 cases were reported on the 24th of February 2020 as passengers disembarking a plane from Iran tested positive [13]. Kuwait responded to this crisis with early aggressive public health interventions such as school and airport closures, curfews, and restrictions on public gatherings, including group prayers at mosques [14]. The Kuwaiti Ministry of Health also designated the recently-constructed Jaber Al-Ahmad Al-Sabah Hospital as the nation’s official “COVID-19 facility”, with the first roughly 1000 cases of COVID-19 being admitted to this large, well-equipped healthcare facility. Despite these decisive measures, the disease continued to spread, with the migrant labor community being especially adversely affected [8].

Due to this unique demographic profile of Kuwait and the Gulf States, recent studies have demonstrated a health disparity resulting in worse health outcomes for non-Kuwaitis due to heat exposure and air pollution [15, 16]. However, there is limited evidence on possible differences between Kuwaiti and non-Kuwaiti populations in other scenarios. This study aims to explore whether there is a significant difference in health outcomes between non-Kuwaiti and Kuwaiti patients diagnosed with COVID-19.

## Methods

### Study population

We used a prospective COVID-19 registry of all patients in Kuwait who tested positive from February 24th, 2020 to April 20th, 2020, collected from Jaber Al-Ahmad Al-Sabah Hospital, the officially-designated COVID-19 healthcare facility in the country. The registry includes all patients who were admitted to the hospital. To be admitted, patients had to meet one criterion only - having a positive reverse transcriptase polymerase chain reaction (RT-PCR) test result from a nasopharyngeal swab. This means that our registry includes all confirmed patients in Kuwait, whether symptomatic or asymptomatic, since all PCR positive cases were admitted for quarantine, observation and treatment. The study received ethical approval from the Kuwait Ministry of Health’s ethics committee for the protection of human subjects.

### Nationality

We determined the nationality of every COVID-19 patient in the registry from their passports or national Civil ID cards (an official form of identification for all national residents and expatriates that includes a unique identifying number, issued by the Public Authority for Civil Information).

### Outcomes

Patients are kept in the hospital and will only be discharged if they: 1) last tested positive 14 days or more, 2) no respiratory symptoms for 7 days, 3) afebrile for more than 3 days, 4) negative PCR test result, 5) improving imaging. All swabs, baseline tests and imaging were performed at the Jaber Al-Ahmad Al-Sabah Hospital laboratory. Each patient on the registry was followed for 5 possible outcomes (not mutually exclusive): in-hospital death, admission to an intensive care unit (ICU), a diagnosis of acute respiratory distress syndrome (ARDS), a diagnosis of pneumonia, and discharge from hospital care. Patients who did not have any documentation of these outcomes, i.e. are still receiving treatment or under observation/quarantine with no adverse outcome nor discharge to our administrative end date (April 20, 2020), were excluded from the study. ARDS was defined using the WHO interim guidance on clinical management of severe acute respiratory infection when COVID-19 is suspected [17]. Diagnosis of pneumonia was defined using American Thoracic Society and Infectious Diseases Society of America [18].

### Covariates

We collected the following baseline characteristics: age, gender, body mass index (BMI), and smoking history (current smoker vs. not a current smoker). We also collected information on patients’ self-reported comorbidities as stated upon history-taking. We specifically documented the presence of the following diseases (Yes/No): diabetes mellitus, hypertension, coronary artery disease/ischemic heart disease, asthma, chronic obstructive pulmonary disease, cerebrovascular disease, hepatitis, dyslipidemia, history of cancer, hypothyroidism, chronic renal disease, immunodeficiency, recent surgery (during the past 30 days) and ongoing pregnancy. If there were comorbidities other than the ones we prespecified, they were documented as ‘other diseases’ (Yes/No).

### Statistical analysis

All continuous variables were summarized using mean, standard deviation, median, minimum and maximum value. All categorical variables were summarized using number and percentage. We ran separate logistic regression models for the following outcomes; death, ICU admission, ARDS and pneumonia. We also used a composite outcome of either death or ICU admission. Assuming a rare event where odds ratios approximate relative probabilities, we reported the odds ratios of each outcome comparing non-Kuwaitis to Kuwaitis. Choice of covariate inclusion in the models to control for confounding was based on a priori clinical hypotheses and preserving low variability in estimating coefficients. We created three models; model 1 is adjusted for age, smoking and a priori selected comorbidities: hypertension, diabetes, cardiovascular disease, asthma, and cancer; model 2 is adjusted for all variables in model 1 and gender; and model 3 is adjusted for all variables in model 2 and body mass index. We also stratified the analysis by gender to examine effect measure modification. In a sensitivity analysis, we created a variable that sums all the measured comorbidities (unweighted) for each individual and adjusted for it as a continuous covariate. Alpha level was set at 0.05 and all analyses were done using R statistical software version 3.4.3 (R Foundation for Statistical Computing, Vienna, Austria).

## Results

The first 1123 COVID-19 positive patients in Kuwait from February 24th to April 20th 2020, were all recruited in this study. All patient characteristics are shown in Table 1. About 26% (*n* = 294) were Kuwaitis and 73% (*n* = 829) were non-Kuwaiti. The largest difference between these two groups was in gender composition. The Kuwaiti group had roughly an equal number of females and males with 47% (*n* = 140) and 52% (*n* = 154) respectively, while the non-Kuwaiti group had a majority of 92% (*n* = 759) composed of males with only 8% of the group being females (*n* = 70).

**Table 1:** Characteristics of the first 1,123 hospitalized patients with COVID-19 on admission in Kuwait

	Kuwaiti (***N*** = 294)	Non-Kuwaiti (***N*** = 829)	Overall (***N*** = 1123)
**Gender**			
Female	140 (47.6%)	70 (8.4%)	210 (18.7%)
Male	154 (52.4%)	759 (91.6%)	913 (81.3%)
**Age (years)**			
Mean (SD)	44.3 (19.2)	41.0 (12.5)	41.9 (14.6)
Median [Min, Max]	45.0 [1.00, 93.0]	40.0 [2.00, 87.0]	40.0 [1.00, 93.0]
**Body Mass Index (kg/m**^**2**^**)**			
Mean (SD)	28.6 (7.08)	26.1 (4.34)	26.8 (5.33)
Median [Min, Max]	27.0 [14.0, 50.0]	26.0 [14.4, 45.0]	26.0 [14.0, 50.0]
Missing	107 (36.4%)	315 (38.0%)	422 (37.6%)
**Smoker**			
Non-Smoker	281 (95.6%)	798 (96.3%)	1079 (96.1%)
Smoker	13 (4.4%)	31 (3.7%)	44 (3.9%)
**Systolic BP (mmHg)**			
Mean (SD)	126 (16.8)	128 (16.6)	128 (16.7)
Median [Min, Max]	125 [84.0, 180]	127 [80.0, 202]	127 [80.0, 202]
Missing	7 (2.4%)	4 (0.5%)	11 (1.0%)
**Diastolic BP (mmHg)**			
Mean (SD)	76.2 (9.18)	79.6 (10.6)	78.7 (10.3)
Median [Min, Max]	77.0 [47.0, 108]	80.0 [40.0, 137]	80.0 [40.0, 137]
Missing	7 (2.4%)	4 (0.5%)	11 (1.0%)
**Respiratory Rate (breaths per minute)**			
Mean (SD)	20.9 (2.64)	21.0 (2.54)	21.0 (2.57)
Median [Min, Max]	20.0 [0, 40.0]	20.0 [12.0, 55.0]	20.0 [0, 55.0]
Missing	3 (1.0%)	6 (0.7%)	9 (0.8%)
**Heart Rate (beats per minute)**			
Mean (SD)	84.8 (12.8)	86.7 (12.8)	86.2 (12.8)
Median [Min, Max]	82.0 [60.0, 136]	86.0 [37.0, 130]	85.0 [37.0, 136]
Missing	2 (0.7%)	4 (0.5%)	6 (0.5%)
**Temperature (degree C)**			
Mean (SD)	36.8 (0.414)	37.0 (0.542)	36.9 (0.517)
Median [Min, Max]	36.8 [35.6, 38.8]	36.9 [35.2, 39.8]	36.8 [35.2, 39.8]
Missing	1 (0.3%)	5 (0.6%)	6 (0.5%)

Table 2 presents all comorbidities seen amongst the two groups. On average, about half (48%) of the Kuwaiti group had at least one co-morbidity and about a quarter (26%) of the non-Kuwaiti group had at least one comorbidity. The most common morbidity in both Kuwaiti and non-Kuwaiti groups was hypertension, with 17% of the entire group suffering from hypertension, although this condition was more prevalent in the Kuwaiti group (28%), as compared to the non-Kuwaiti group (13%). The second most common illness was diabetes mellitus, which was self-reported in 15% of the entire group, while still more common in the Kuwaiti group (23%) in comparison with non-Kuwaitis (12%). Finally, 4% of the group had asthma, with a higher percentage in the Kuwaiti (9%) group than non-Kuwaiti (3%) group.

**Table 2:** Co-Morbidities of 1,123 Patients Hospitalized With COVID-19

	Kuwaiti (***N*** = 294)	Non-Kuwaiti (***N*** = 829)	Overall (***N*** = 1123)
**Any comorbidity**			
At least one comorbidity	140 (47.6%)	214 (25.8%)	354 (31.5%)
No comorbidities	154 (52.4%)	615 (74.2%)	769 (68.5%)
**Hypertension**			
No	213 (72.4%)	721 (87.0%)	934 (83.2%)
Yes	81 (27.6%)	108 (13.0%)	189 (16.8%)
**Diabetes**			
No	227 (77.2%)	728 (87.8%)	955 (85.0%)
Yes	67 (22.8%)	101 (12.2%)	168 (15.0%)
**Asthma**			
No	269 (91.5%)	806 (97.2%)	1075 (95.7%)
Yes	25 (8.5%)	23 (2.8%)	48 (4.3%)
**Total Comorbidity**^**a**^			
0	154 (52.4%)	615 (74.2%)	769 (68.5%)
1	55 (18.7%)	132 (15.9%)	187 (16.7%)
2	38 (12.9%)	57 (6.9%)	95 (8.5%)
3	30 (10.2%)	19 (2.3%)	49 (4.4%)
4	14 (4.8%)	6 (0.7%)	20 (1.8%)
5	2 (0.7%)	0 (0%)	2 (0.2%)
6	1 (0.3%)	0 (0%)	1 (0.1%)

^a^Comorbidities were diabetes mellitus, hypertension, coronary artery disease/ischemic heart disease, chronic obstructive pulmonary disease, asthma, cerebrovascular disease, hepatitis, dyslipidemia, cancer, hypothyroidism, chronic renal disease, immunodeficiency, recent surgery (during the past 30 days), pregnancy, and other

**Fig. 1:**
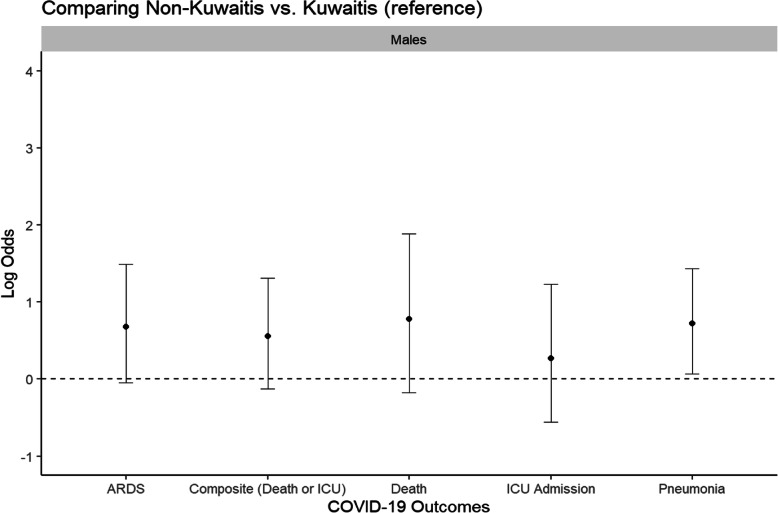
Log odds of COVID-19 outcomes comparing non-Kuwaitis to Kuwaitis among males (adjusted for age, smoking and comorbidities (hypertension, diabetes mellitus, cardiovascular disease, asthma and cancer)

Table 3 represents COVID-19 outcomes stratified by nationality in the study population. Table 4 represents adjusted associations between nationality and adverse outcomes, with Kuwaitis as the reference group. With adjustments made to age, gender, smoking and selected co-morbidities, non-Kuwaitis had nearly two-fold increase in the odds of being admitted to the intensive care unit compared to Kuwaitis (OR: 1.92, 95% CI 0.86–4.72). The odds ratios of death were also two times higher than Kuwaitis (OR: 2.19, 95% CI 0.88–6.02). Furthermore, non-Kuwaitis had also higher odds of ARDS (OR:2.44, 95% CI 1.23–5.09) and pneumonia (OR: 2.24, 95% CI 1.27–4.12). When adjusting for gender, both admission to the intensive care unit and death did not show statistically significant results. However, when both outcomes were combined, non-Kuwaitis were two times more likely to get these adverse outcomes (OR: 2.14, 95% CI 1.12–4.32). When adjusted to body mass index (which was missing in 442 individuals), no outcomes provided statistically significant results, yet the direction of effects remained the same. Sensitivity analysis adjusting for comorbidity scores showed the same inference (results not shown). Figure 1 shows effect estimates for the different outcomes comparing non-Kuwaitis to Kuwaitis while restricting the analysis to males only.

**Table 3:** Clinical Outcomes of 1,123 Patients Hospitalized With COVID-19

	Kuwaiti (***N*** = 294)	Non-Kuwaiti (***N*** = 829)	Total (***N*** = 1123)
**Admission to ICU**			
Admitted	11 (3.7%)	40 (4.8%)	51 (4.5%)
Not Admitted	283 (96.3%)	789 (95.2%)	1072 (95.5%)
**Death**			
Death	10 (3.4%)	30 (3.6%)	40 (3.6%)
No Death	284 (96.6%)	799 (96.4%)	1083 (96.4%)
**Composite Outcome (ICU or Death)**			
Yes	21 (7.1%)	70 (8.4%)	91 (8.1%)
No	273 (92.9%)	759 (91.6%)	1032 (91.9%)
**In-hospital ARDS**			
In-hospital ARDS	20 (6.8%)	64 (7.7%)	84 (7.5%)
No ARDS	274 (93.2%)	765 (92.3%)	1039 (92.5%)
**Pneumonia**			
No Pneumonia	264 (89.8%)	736 (88.8%)	1000 (89.0%)
Pneumonia	30 (10.2%)	93 (11.2%)	123 (11.0%)

**Table 4:** Odds ratios of COVID-19 outcomes comparing non-Kuwaitis to Kuwaitis

	Odds Ratio	Lower CI	Upper CI	***p***-value
**ICU Admission**				
Model 1	2.434	1.161	5.557	0.025
Model 2	1.926	0.856	4.723	0.131
Model 3	1.930	0.770	5.483	0.186
**Death**				
Model 1	3.463	1.463	9.082	0.007
Model 2	2.190	0.884	6.019	0.108
Model 3	1.627	0.535	5.915	0.422
**Composite Outcome (Death or ICU)**				
Model 1	3.099	1.693	5.972	< 0.001
Model 2	2.143	1.117	4.316	0.027
Model 3	1.902	0.882	4.412	0.116
**ARDS**				
Model 1	3.297	1.752	6.552	< 0.001
Model 2	2.439	1.229	5.091	0.014
Model 3	1.899	0.850	4.592	0.134
**Pneumonia**				
Model 1	2.626	1.556	4.593	< 0.001
Model 2	2.241	1.265	4.108	0.007
Model 3	1.456	0.756	2.918	0.274

Model 1: adjusted for age, smoking and comorbidities (hypertension, diabetes mellitus, cardiovascular disease, asthma and cancer

Model 2: adjusted for variables in Model 1 plus gender; Model 3: adjusted for variables in Model 2 plus BMI

## Discussion

This is the first study to report on COVID-19 outcomes between Kuwaiti and non-Kuwaiti patients. We found non-Kuwaitis were two times more likely to be admitted to the ICU or die from COVID-19. With adjustment to age, smoking and certain co-morbidities, non-Kuwaitis had two- and three-fold increase in odds ratios for ICU admission and death, respectively. While in many instances our underpowered results did not show statistical significance, the effect estimates for all outcomes were consistently in the same direction and magnitude especially when we considered a composite outcome of ICU admission or death.

Ethnicity is a complex entity. It represents social constructs, cultural identities, behaviors, as well as a large pool of genetic make-up [19]. The non-Kuwaiti subpopulation is a mixture of different ethnicities and races. We therefore interpret our findings in terms of socioeconomic and environmental rather than biological explanations. In Kuwait, migrant workers make up the majority of non-Kuwaitis. These workers usually have poor working and housing conditions and often performing unskilled labor jobs with minimal formal education. Most migrant workers are employed in unskilled labor jobs with low-paying wages and a background of low socioeconomic status (SES) [20, 21]. Hence, the non-Kuwaiti subpopulation can serve as a proxy for SES.

Healthcare access and use may have a role in explaining the health disparities among marginalized subpopulations.^4^ For one, non-Kuwaitis might have underlying medical conditions that were left undiagnosed and untreated. Such conditions might be key factors into making their condition with COVID-19 worse. In general, migrant workers may have difficulty communicating with medical professionals, do not have access to interpreters and have limited knowledge of the health insurance systems [21, 22]. Kuwait has passed laws and regulations ensuring that all COVID-19 related care is provided for free regardless of citizenship status. However, with regards to non-COVID-19 care, all public-sector healthcare is provided to Kuwaiti citizens for free, but there may be limitations on coverage for non-Kuwaiti expatriates, with basic healthcare services being covered by an annual health insurance fee. Similarly, ineffective public health messaging with migrant workers who grapple with linguistic and cultural barriers may delay diagnosis and treatment of COVID-19 [9, 23]. That is, non-Kuwaiti migrant workers may be presenting late and with more severe symptoms.

It is noteworthy that in studies that adjusted for socioeconomic factors, variations in hospitalization and mortality in marginalized subpopulations were not fully explained [23, 24]. Therefore, while our socioeconomic explanations are tentative, other differences including the environment warrant careful examination. Migrant workers in particular tend to live in over-crowded housing with unsanitary, shared bathrooms and kitchens. The segregated unmaintained residential neighborhoods that they live in may be another contributor to poor health [25, 26]. Many are living in food deserts, and their meager wages - most of which are sent overseas to support their families - may leave unhealthy food outlets as the only affordable option. Limited local transportation in such neighborhoods, or an inability for individuals to afford access to personal transportation may further frustrate their access to healthcare. Migrant workers in Kuwait were especially vulnerable to air pollution and extreme heat owing to a significant high exposure heterogeneity among the population in Kuwait [15, 16]. Emerging research is now showing that people living in areas with high outdoor ambient air pollution levels were at higher risk of dying from COVID-19 compared to those living in less polluted neighborhoods [27]. It is possible that the long-term exposure to air pollution may play a role in exacerbating the severity COVID-19 infections among non-Kuwaitis.

It may be presumptuous to think that these findings alone will be able to offer solutions to the multitude of issues that lead to the poor health of migrant workers in Kuwait. Yet, in the short term, whilst we deal with this pandemic, the job of active surveillance in marginalized communities should continue. The bases of test, trace and isolate is as important here as in any subpopulation, as these are the measures that have been shown time and time again to slow the spread of COVID-19. Fundamentally however, to improve migrant worker’s health, Kuwait must begin by improving the general working and living conditions for migrant labor.

Suggestions of genetic susceptibility among ethnic minorities and COVID-19 outcomes are at best inconclusive and at worst could lead to victim-blaming. For example, different people may have different ACE2 (the receptor for SARS-CoV-2) expressions leading to greater susceptibility to COVID-19 in some subgroups [28]. One study used single-cell sequencing, reported that expression of ACE2 was more predominant in Asian men [29]. An observational study looked at patients in two Spanish hospitals and found an increased prevalence of androgenetic alopecia amongst those infected with COVID-19 in which no hormonal measures were taken [30]. Taken together, the link is not yet established, and further studies need to be conducted before such conclusions can be taken.

This study has a number of limitations. Firstly, being non-Kuwaiti (our exposure of interest) is not necessarily a valid proxy of low SES for every non-Kuwaiti individual. Since we did not have SES data, we may have included individuals with high SES resources under the non-national variable. However, if we assume that high SES is associated with a lower probability of adverse COVID-19 outcomes, then the misclassification bias from this proxy may have attenuated the relationship we observed. Secondly, control for smoking was not optimal. The prevalence of smoking in our study population was very low and we did not have information on long-term use (e.g. pack-years, or years of smoking). Although the evidence is still not conclusive, if smoking is associated with a higher probability of adverse COVID-19 outcomes and is more prevalent amongst the non-Kuwaiti sub-population, then we cannot rule out a residual confounding that could overestimate our observed relationship. Thirdly, many individuals had missing BMI data. In our complete-case-only analysis (model 3), the low sample size produced wide variability around our estimates when we adjusted for BMI. Fourth, we did not have data on neighborhood characteristics including air pollution, which is likely to be worse in areas populated by non-Kuwaitis compared to Kuwaitis. If, for example, long-term exposure to air pollution is associated with worse COVID-19 outcomes, then we may have overestimated our observed estimates since we did not control for it. Finally, our sample size was not powered enough to detect interaction by gender.

## Conclusion

The current pandemic could amplify societal and structural inequalities that have long existed in the public health realm. The data on the differences of COVID-19 outcomes among marginalized subpopulations is now growing. In Kuwait we found non-Kuwaitis to be two times more likely to be admitted to the ICU or die from COVID-19. A number of socioeconomic and environmental factors could explain this health disparity. More studies are needed to explore the role of these factors and advance the understanding of policymakers in Kuwait to resolve these health gaps with urgent public health interventions.

## Data Availability

The dataset used for the current study is not publicly available due to the Ministry of Health protection from possible recognition of a patient. For access of data, please contact: Dr. Salman Al Sabah: salman.k.alsabah@gmail.com

## Abbreviations

ACE-2: Angiotensin-converting enzyme 2
ARDS: Acute respiratory distress syndrome
BMI: Body mass index
COVID-19: Coronavirus disease 2019
ICU: Intensive care unit
OR: Odds ratio
PCR: Polymerase chain reaction
SES: Socio-economic class
WHO: World health organization
